# The effect of single nucleotide polymorphisms on depression in combination with coronary diseases: a systematic review and meta-analysis

**DOI:** 10.3389/fendo.2024.1369676

**Published:** 2024-04-30

**Authors:** Jing Zhang, Lu Gao, Guan Lin Yang, De Zhao Kong

**Affiliations:** Liaoning University of Traditional Chinese Medicine, Shenyang, Liaoning, China

**Keywords:** depression, coronary heart disease, gene polymorphisms, single nucleotide polymorphisms, systematic review, meta-analysis

## Abstract

**Background:**

Depression and coronary heart disease (CHD) have common risk mechanisms. Common single nucleotide polymorphisms (SNPs) may be associated with the risk of depression combined with coronary heart disease.

**Methods:**

This study was designed according to the PRISMA-P guidelines. We will include case-control studies and cohort studies investigating the relationship between gene SNPs and depression and coronary heart disease comorbidities. The Newcastle-Ottawa Scale (NOS) will be used to assess the risk of bias. When measuring dichotomous outcomes, we will use the odds ratio (OR) and 95% confidence interval (95%CIs) in a case-control study. Five genetic models (allele model, homozygous model, co-dominant model, dominant model, and recessive model) will be evaluated for each included study. Subgroup analysis by ethnicity will be performed. If necessary, *post hoc* analysis will be made according to different types.

**Results:**

A total of 13 studies were included in this study, and the types of genes included are FKBP5 and SGK1 genes that act on glucocorticoid; miR-146a, IL-4-589, IL-6-174, TNF-α-308, CRP-717 genes that act on inflammatory mechanisms; eNOS genes from endothelial cells; HSP70 genes that act on the autoimmune response; ACE2 and MAS1 genes that act to mediate Ang(1-7) in the RAS system; 5-HTTLPR gene responsible for the transport of serotonin 5-HT and neurotrophic factor BDNF gene. There were three studies on 5-HTTLPR and BDNF genes, respectively, while there was only one study targeting FKBP5, SGK1, miR-146a, IL-4-589, IL-6-174, TNF-alpha-308, CRP-717, eNOS, HSP70, ACE2, and MAS1 genes. We did not perform a meta-analysis for genes reported in a single study, and meta-analysis was performed separately for studies exploring the 5-HTTLPR and BDNF genes. The results showed that for the 5-HTTLPR gene, there was a statistically significant association between 5-HTTLPR gene polymorphisms and depression in combination with coronary diseases (CHD-D) under the co-dominant model (LS vs LL: OR 1.76, 95%CI 1.20-2.59; SS vs LL: OR 2.80, 95%CI 1.45 to 5.41), the dominant model (LS+SS vs LL: OR 2.06, 95%CI 1.44 to 2.96), and the homozygous model (SS vs LL: OR 2.80 95%CI 1.45 to 5.5.41) were statistically significant for CHD-D, demonstrating that polymorphisms in the 5-HTTLPR gene are associated with the development of CHD-D and that the S allele in the 5-HTTLPR gene is likely to be a risk factor for CHD-D. For the BDNF gene, there were no significant differences between one of the co-dominant gene models (AA vs GG: OR 6.63, 95%CI 1.44 to 30.64), the homozygous gene model (AA vs GG: OR 6.63,95% CI 1.44 to 30.64), the dominant gene model (GA+AA vs GG: OR4.29, 95%CI 1.05 to 17.45), recessive gene model (AA vs GG+GA: OR 2.71, 95%CI 1.16 to 6.31), and allele model (A vs G: OR 2.59, 95%CI 1.18 to 5.67) were statistically significant for CHD-D, demonstrating that BDNFrs6265 gene polymorphisms are associated with the CHD-D development and that the A allele in the BDNFrs6265 gene is likely to be a risk factor for CHD-D. We analyzed the allele frequencies of SNPs reported in a single study and found that the SNPs in the microRNA146a gene rs2910164, the SNPs in the ACE2 gene rs2285666 and the SNPs in the SGK1 gene rs1743963 and rs1763509 were risk factors for the development of CHD-D. We performed a subgroup analysis of three studies involving the BDNFrs6265 gene. The results showed that European populations were more at risk of developing CHD-D than Asian populations in both dominant model (GA+AA vs GG: OR 10.47, 95%CI 3.53 to 31.08) and co-dominant model (GA vs GG: OR 6.40, 95%CI 1.98 to 20.73), with statistically significant differences. In contrast, the studies involving the 5-HTTLPR gene were all Asian populations, so subgroup analyses were not performed. We performed sensitivity analyses of studies exploring the 5-HTTLPR and BDNF rs6265 genes. The results showed that the results of the allele model, the dominant model, the recessive model, the homozygous model and the co-dominant model for both 5-HTTLPR and BDNF rs6265 genes were stable. Due to the limited number of studies of the 5-HTTLPR and BDNF genes, it was not possible to determine the symmetry of the funnel plot using Begg’s funnel plot and Egger’s test. Therefore, we did not assess publication bias.

**Discussion:**

SNPs of the microRNA146a gene at rs2910164, the ACE2 gene at the rs2285666 and the SGK1 gene at rs1743963 and rs1763509, and the SNPs at the 5-HTTLPR and BDNF gene loci are associated with the onset of comorbid depression in coronary heart disease. We recommend that future research focus on studying SNPs’ impact on comorbid depression in coronary heart disease, specifically targeting the 5-HTTLPR and BDNF gene at rs6265.

**Systematic review registration:**

https://www.crd.york.ac.uk/prospero/, identifier CRD42021229371.

## 1 Introduction

Depression is a common mental disorder. According to WHO reports, more than 264 million people suffer from depression in global terms (1). Depression and coronary heart disease (CHD) are leading causes of disability and disease burden in high-income countries (2). So far, many meta-analyses and reviews have proved that depression has a strong correlation with the increase in the incidence and mortality of CHD (3–9). The World Mental Health Survey results showed that cardiac patients have twice the risk of depression than those without heart disease (10). In 2006, Thombs et al. (11) found the probability of patients with myocardial infarction suffering from depression is between 15.5% and 31.1%. In 2007, Egede et al. (12) found the prevalence of major depressive disorder (MDD) in cardiac patients is 9.3%, while it is 4.8% in no comorbidity individuals. Depression can adversely affect the prognosis of CHD patients (13). Depressed patients are challenged to comply with medical treatment (14). Ziegelstein et al. (15) raised that depressed myocardial infarction patients should follow recommendations to reduce heart risk difficulty during the recovery period. Lichtman et al. (16) proved that high levels of biomarkers predicting cardiac events or promoting atherosclerosis are found frequently in people with depression.

CHD and mental diseases’ common risk mechanisms include endothelial cell and platelet dysfunction, inflammation, autonomic dysfunction, and hypothalamus-pituitary-adrenal cortex (HPA) axis dysfunction (17). Researchers put forward the concept of “gene overlap” based on these common risk mechanisms, meaning the involvement of the same genes in the pathogenesis of both CHD and depression (18).

5-HTT (5-hydroxytryptamine transporter) is encoded by the SLC6A4 (solute carrier family 6 member 4) gene localized in chromosome 17q11.1-q12 (19) and expressed in brain and blood cells. The pathophysiological mechanism of depression may be associated with an imbalance of 5-HT uptake in the synaptic cleft mediated by 5-HT transporter (20, 21). Besides, alterations of 5-HT mechanisms may be related to developing an enhanced cardiovascular risk (22, 23). Galan et al. (24) showed that 5-HT is an agonist of platelets in peripheral tissues. It enhances the procoagulant response and increases thrombogenesis on damaged vascular surfaces. Some meta-analyses and prospective studies have concluded that 5-HT transporter linked promoter region (5-HTTLPR) polymorphisms may significantly impact the risk of depression in CHD patients (25–29). Phillips et al. (30) found that patients with depression who carry the L allele in patients after coronary artery bypass graft surgery in the United States were more likely to have adverse events; Nakatani et al. (31) showed that the risk of depression and cardiac adverse events in patients with acute myocardial infarction in Japan during the recovery period is related to the S allele; Kim et al. (32) proposed that Koreans carrying the S allele are related to the occurrence of post-acute coronary syndrome (ACS) depression.

Dysfunctional serotonin 2A receptor (5-HT2AR) and serotonin 2C receptor (5-HT2CR) are implicated in neuropsychiatric disorders (33). As one of the main pharmacological therapeutic targets for MDD, 5-HT2AR has a high affinity for antidepressants (34). The 5-HT2CR antagonist is a commonly used drug for the treatment of significant depression (35). In a case-control study conducted in Russia by Golimbet et al. (36), they found that 5-HTR2A polymorphism -1438A/G is related to the severity of depressive symptoms in CHD patients, and the risk of moderate and severe depression in patients with allele G is 2.4 times higher than that in patients with genotype AA. The 5-HTR2C polymorphism Cys23Ser is associated with depression, and Ser alleles have a higher incidence in CHD patients (36).

Apelin (APLN), an endogenous neuropeptide, is the cognate ligand for the G protein-coupled receptor APJ (putative receptor protein related to angiotensin II receptor type-1, AT1R) (37). Apelin/APJ system plays a potential role in emotional behavior (38). However, the role of apelin in depression is controversial (39). In the cardiovascular system, the apelin-APLNR pathway plays a central role, and circulating apelin is a promising CHD predictor (40). Wang et al. (41) conducted a case-control study of 269 patients with CHD (122 of them suffering from depression) and 184 healthy people in China. It is the first report that after adjusting drinking habits, insomnia, hypertension, and stroke history, patients with CHD who carry the APLNR rs9943582 C allele still have a higher risk of depression.

Brain-derived neurotrophic factor (BDNF) regulates vascular development and response to injury by activating local TrkB-expressing endothelial cells(ECs) and inducing mobilization and recruitment of myeloid cells’ subpopulation (42). Lower BDNF levels are associated with the persistence of depressive symptoms in CHD patients (43). The Val66Met polymorphism of the BDNF gene is associated with depression (44). Kang et al. (45) found that Korean ACS patients carrying the BDNF Met allele were related to the prevalence and persistence of depression. Bozzini et al. (25) found that the BDNF AA genotype is involved in the pathogenesis of CHD in women and the susceptibility of CHD related to depression in a case-control study involving 99 CHD patients and 143 healthy people in Italy. Liu et al. (46) found a significant correlation between CHD with depression and the SNP rs6265 located in the fourth exon of the BDNF gene and a potential correlation with the promoter region rs13306221.

Apolipoprotein E (ApoE) participates in plasma lipoprotein metabolism by interacting with cell surface receptors (47). ApoE prevents atherosclerosis progression (48), and lack of ApoE leads to spontaneous development of atherosclerosis (49). Studies in the population show that ApoE polymorphism is the primary determinant of an individual’s susceptibility to CHD (50). ApoE is also involved in the process of nervous system growth and regeneration after injury (47). Ji et al. (51) included a case-control study of 30 CHD patients, 26 CHD patients with depression and 30 healthy people in China, which showed that the ApoEϵ4 allele might play an important role in depression in combination with CHD.

FK-506 binding protein 51 (FKBP5) is a co-chaperone of heat shock protein 90 (hsp90). A complex of Hsp90 and FKBP51 slows down glucocorticoid receptor (GR) transport into the nucleus and reduces GR’s activity (52), which leads to the weakening of GR’s negative feedback on the HPA axis. HPA axis is the central stress hormone system and is linked with the development of CHD and depression when exposed to stressors (53, 54). FKBP5 might confer a shared genetic risk for both CHD and depression (55). Brandt et al. (55) included a prospective study of 268 German CHD patients and found that depression was only associated with the FKBP5 rs1360780 C allele in patients with previous myocardial infarction or coronary artery reconstruction. Wang et al. (56) included a case-control study of 271 CHD patients (123 of them with depression) and 113 healthy controls from the Han nationality in northern China. They found that rs9470079 may be a potential gene locus of co-morbidity of CHD and depression.

Glucocorticoid receptor (GR) is a steroid hormone receptor, which belongs to the nuclear receptor superfamily of transcription factors (57). It is highly expressed in the HPA axis’s critical regions, including the hippocampus, amygdala, and hypothalamus (58). As a negative feedback mechanism of the HPA axis, GR in the hypothalamus and pituitary gland binds to cortisol, inhibits ACTH and CRH’s secretion, and regulates the homeostasis of the HPA axis (59). More and more studies have verified that GR dysfunction is involved in the pathological mechanism of depression and depressive behavior caused by stress (60–62). Over the past few decades, many researchers have confirmed a causal relationship between glucocorticoid receptor gene (Nuclear receptor subfamily 3 group C member1, NR3C1) SNPs depression (63–65). Currently, the relationship between glucocorticoids and atherosclerosis is complicated and unclear. A review pointed out that GR’s chronic excessive activation induces cardiovascular risk factors, such as obesity, insulin resistance, glucose, intolerance, dyslipidemia, and hypertension (66). NR3C1 polymorphism may affect the sensitivity of cells to glucocorticoids by changing the transcription level of NR3C1, affecting the number of receptors or affinity of hormones and receptors, thus leading to individual dependence or resistance to glucocorticoids (67). Otte et al. (68) tested four NR3C1 gene polymorphism types (ER22/23EK, BclI C/G, n363, and 9beta A/G) in a cross-sectional genetic association study of 526 white American patients with chronic CHD. The study results indicate that haplotype 3, which contains the minor allele of the 9beta A/G polymorphism, has a gene dose-dependent relationship with depression. Haplotype 3 may be a susceptibility factor for depression in CHD patients.

Serum/glucocorticoid-regulated kinase 1 (SGK1) is a serine/threonine kinase, a member of the AGK Kinase family, and contributes to transport regulation hormone release, neuron excitability, inflammation, cell proliferation, and apoptosis (69). SGK1 participates in renal Na^+^ excretion by aldosterone, insulin, and insulin-like growth factor 1(IGF1) (70, 71), thereby affecting blood pressure. The genetic variance of SGK1 is pertinent to blood pressure (72). SGK1, significantly associated with depression, is a mediator for cortisol effects on neurogenesis and GR function (73). Considering the complicated relationship among SGK1, CHD, and depression, it is reasonable to propose that SGK1 may be a co-pathogenic gene in the comorbid mechanism of CHD and depression. Han et al. (74) tested the SGK1 gene in 257 Han Chinese CHD patients (69 cases of depression) and 107 healthy people. They found that both rs1743963 GG genotype and rs1763509 AA genotype were associated with an increased risk of depression in CHD patients. Haplotype GGA significantly increases the risk of depression in CHD patients, and haplotype AAG may be a protective factor for patients with CHD and depression.

Plasminogen activator inhibitor-1 (PAI-1) is a principal regulatory protein in the fibrinolytic system, as the primary inhibitor of tissue plasminogen activator (tPA) and urokinase plasminogen activators (uPA) (75). Decreased fibrinolytic activity in CHD patients is associated with PAI-1 (76). The plasminogen activator inhibitor-1 gene (*SERPINE1*) is located on chromosome 7 (7q22.1). Evidence suggests that *SERPINE1* genetic variants may play a role in MDD and CHD susceptibility (75, 77). In a study covering 42 depressed patients, 65 CHD patients, and 132 healthy people in China, Lin et al. (78) found that the frequency of PAI-1 gene -675 locus 4G/4G gene and 4G allele in depressed patients and CHD patients are higher than in the healthy control group. There is no significant difference between the 4G/4G genotype frequency and the 4G allele frequency. Therefore, PAI-1 gene 4G may be a comorbid gene of CHD and depression. Xia et al. (79) included 75 CHD patients with depression, 91 CHD patients, 56 patients with depression, and 63 healthy people. The study found that the PAI-1 gene 4G/4G and 4G allele frequency in CHD patients and CHD with depression was significantly higher than that of the other two groups. It also shows that the co-morbidity of CHD and depression is related to the coexistence of the 5-HTTLPR gene SS genotype and PAI-1 gene 4G/4G genotype.

However, the association between gene polymorphisms and depression in combination with CHD is controversial. Up till now, a high-quality, comprehensive systematic review of possible gene SNPs on depression in combination with CHD has not been conducted or published. This study will systematically review the correlation between depression in combination with CHD and SNPs using meta-analysis.

## 2 Methods

This study was performed complying with the Preferred Reporting Items for Systematic Review and Meta-Analysis Protocols (PRISMA-P) and published on the International Prospective Register of Systematic Reviews(PROSPERO) on 10 January 2021. It will last update on 20 April 2021 (registration number CRD42021229371) (80).

### 2.1 Literature search

#### 2.1.1 Information sources

We will search Cochrane Central Register of Controlled Trials (CENTRAL) in The Cochrane Library, MEDLINE Ovid, Embase Ovid, Science Citation Index Expanded (Web of Science), and also search for the China National Knowledge Infrastructure (CNKI), Chongqing VIP (CQVIP), China Biomedical Literature Service System (SinoMed) and Wanfang Data. To ensure literature saturation, We will search the Chinese Clinical Trial Registry (ChiCTR) for ongoing or unpublished studies and search in Human Genomic Epidemiology Navigator (HuGENavigator) based on the genes retrieved from the above database. We manually searched reference lists of systematic reviews and meta-analyses on this topic and retrieved studies.

#### 2.1.2 Search strategy

We will use medical subject headings (MeSH) to develop literature search strategies. The search was first performed using “polymorphism or mutation or variant” as the medical subject term.

We searched MEDLINE Ovid up to 10 January 2021 for phrases:

1 (polymorphism or mutation or variant).sh. [sh = MeSH subject heading].
2 (polymorphism or mutation or variant).mp. [mp=title, abstract, heading word, drug trade name, original title, device manufacturer, drug manufacturer, device trade name, keyword, floating subheading word].
3 depression.sh.
4 (depression or depressive).mp.
5 coronary disease.sh.
6 (heart disease or cardiovascular disease or coronary artery disease or coronary atherosclerosis or angina pectoris or pectoris or acute coronary syndrome or myocardial infarction or myocardial ischemia or ischemic heart disease or CHD or CAD or CVD or ACS or MI).mp.
7 1 or 2.
8 3 or 4.
9 5 or 6.
10 7 and 8 and 9.

### 2.2 Inclusion criteria

#### 2.2.1 Participants

The case group in the study included patients with a diagnosis of depression combined with CHD, as defined by the trialists or according to guidelines, and control group participants included patients with a diagnosis of CHD that did not include depression or patients with a diagnosis of depression alone. This study’s CHD includes angina, acute coronary syndrome, ischemic heart disease, and myocardial infarction — no requirement on age and gender.

#### 2.2.2 Exposure

Any SNPs associated with coronary heart disease combined with depression will be searched. This includes genes such as 5-HTT, BDNF, FKBP5, SGK1, eNOS, miR-146a, HSP70, ACE2, MAS1, IL-4-589, IL-6-174, TNF-α-308, CRP-717. We will not limit the genotypes or polymorphic mutation types retrieved, but the studies retrieved should state the effect of the gene SNPs on coronary heart disease or depression.

#### 2.2.3 Comparator

The polymorphic mutation types of the genotypes in the case group.

#### 2.2.4 Outcome

The proportion of participants with depression combined with coronary diseases.

#### 2.2.5 Types of study to be included

We will include case-control studies and cohort studies investigating the relationship between gene SNPs and depression and CHD comorbidities. The blinding, language, year, publication format, and publication status will be irrespective.

### 2.3 Exclusion criteria

We will use freely available online translators to translate eligible studies in any other language into English. If a translation of the article is unclear, we will contact the original authors by email. If no response is obtained after one month, we will exclude the article. We only include peer-reviewed studies published in scientific journals. Master’s theses, dissertations, abstracts from conference proceedings, technical reports, articles with missing data, and papers where no full text will be excluded.

### 2.4 Data extraction

We have developed consistent screening criteria: 1. We will include case-control studies and cohort studies, and we only include peer-reviewed studies published in scientific journals. 2. The study to investigate the association between genetic polymorphisms and depression combined with coronary heart disease. 3. Raw data, including genotype frequencies, ORs, and 95% CIs, were included in the study. Exclusion criteria: Master’s theses, dissertations, abstracts from conference proceedings, technical reports, articles with missing data, and papers where no full text will be excluded. If a translation of the article is unclear, we will contact the original authors by email. If no response is obtained after one month, we will exclude the article. Review authors conducted separate literature searches using the same search formulas and screened titles and abstracts independently in pairs based on pre-established screening criteria to identify potentially eligible trials, and extracted data using an electronic data collection form created in Microsoft Excel. We resolved any disagreements through discussion, or we asked a third author who was not involved in the data extraction process. Review authors worked in pairs independently extracted the following information: publication data (i.e., year, country, authors); study characteristics and design; characteristics of trial participants; trial diagnostic criteria; the prevalence of SNPs associated with depression combined with coronary disease (mutations detected—original amino acid, mutated amino acid, position, number of carriers of mutated allele in case group and control group, and number of non-carriers of mutated allele in both) among study population were recorded, whether the genotype frequencies conformed to Hardy-Weinberg Equilibrium (HWE), number of dropouts and final number of participants used in the analyses.

### 2.5 Quality assessment

Review authors working in pairs assessed the risk of bias in the included trials. According to the Newcastle-Ottawa Scale (NOS), we will assess the risk of bias about the following items: selection, comparability, and exposure.

### 2.6 Statistical analysis

We will make five comparisons for each polymorphism: 1) allele model (B vs. A), 2) homozygous model (BB vs. AA), 3) co-dominant model (AB vs. AA; BB vs AA), 4) dominant model (BB+AB vs. AA), 5) recessive model (BB vs. AA+AB). We assessed our intervention effects with both fixed-effect model and random-effects model meta-analyses. We reported both results when results differed (e.g., one giving a significant intervention effect, the other no significant intervention effect). We put greater weight on the estimate closest to the zero effect (the highest P-value). We assessed the outcome with a P-value of 0.05 or less as statistically significant. We will use the odds ratio (OR) for measuring dichotomous outcomes with 95% confidence intervals (CIs) for head-to-head comparison meta-analysis for a case-control study and cohort study.

In cases of available data, we will plan to perform the subgroup analyses by ethnicity. If necessary, *post hoc* analysis will be made according to different types, including angina, acute coronary syndrome, ischemic heart disease, and myocardial infarction. We planned to perform sensitivity analyses by omitting single studies from each meta-analysis to assess these studies’ effect on the pooled effect size in the overall model and the subgroup analyses. A study will be omitted if it differes from the other studies based on the pre-specified potentially confounding variables (age, education, ethnicity, history of mental illness). These factors may impact outcomes.

## 3 Results

### 3.1 Literature search and study characteristics

**Figure 1:**
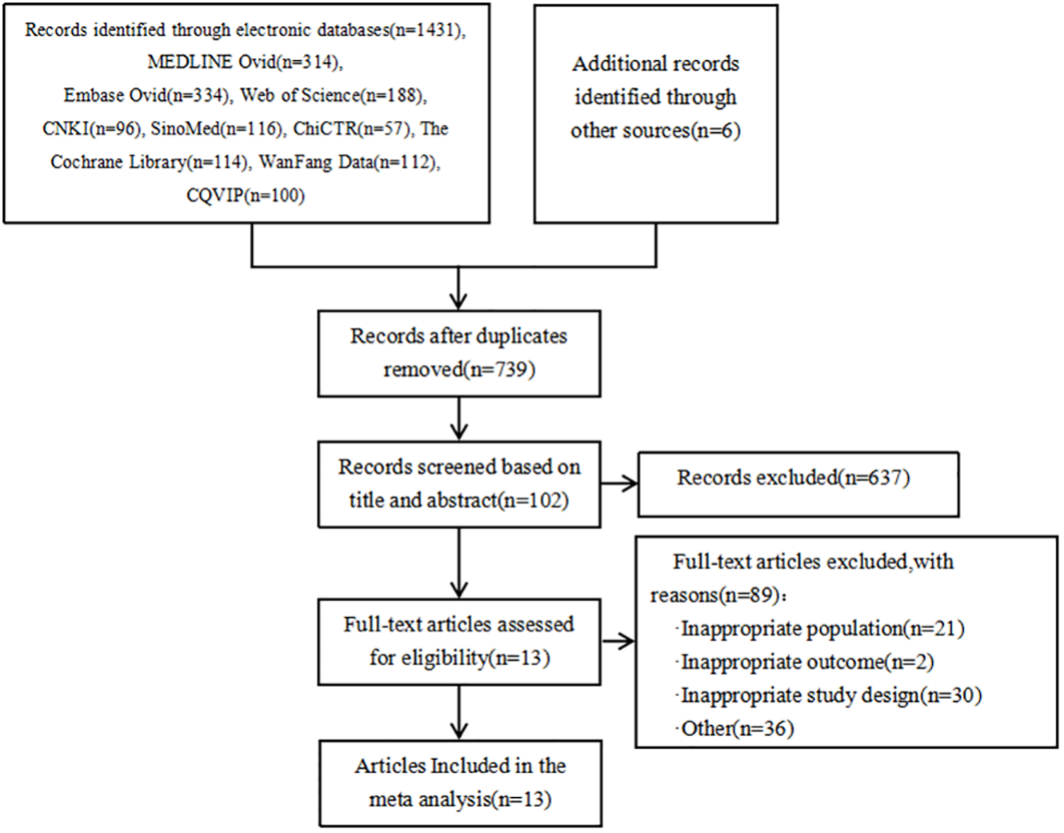
Characteristics of eligible studies.

As shown in **Figure 1**, a total of 1431 articles were detected. Additionally, 6 articles were obtained from other sources. By using the literature management software (NoteExpress), 698 duplicate articles were removed. Based on the titles and abstracts, 637 articles that did not meet the inclusion criteria were excluded. Initially, 102 articles were included. After reading the full text, 89 articles were further excluded, resulting in a final inclusion of 13 articles (25, 31, 46, 56, 74, 79, 81–87). The types of genes included are FKBP5 (88) and SGK1 (89) genes that act on glucocorticoid; miR-146a (90), IL-4-589, IL-6-174, TNF-α-308, CRP-717 genes that act on inflammatory mechanisms (83); eNOS genes from endothelial cells (91); HSP70 genes that act on the autoimmune response (92); ACE2 (93) and MAS1 (94) genes that act to mediate Ang(1-7) in the RAS system; 5-HTTLPR gene responsible for the transport of serotonin 5-HT (29) and neurotrophic factor BDNF gene (95).

The genotype distribution of the control group included in the literature was assessed using the Hardy-Weinberg Equilibrium (HWE) method, the goodness-of-fit test was used to calculate the chi-square value. In the 13 articles, the genotype distribution of the control group for the following genes all conform to the Hardy-Weinberg equilibrium law: eNOS, HSP70, FKBP5, miR-146a, ACE2, MAS1, SGK1, IL-4–589, IL-6–174, TNF-α–308, CRP–717, 5-HTTLPR, and BDNF. The goodness-of-fit test indicates a good fit (P > 0.05). It suggests that the study samples are representative of the population.

The specific characteristics of the included literature are summarized in **Table 1**. The genotyping methods used in the included literature were Polymerase Chain Reaction (PCR), transcription PCR (RT-PCR) technique, Restriction Fragment Length Polymorphism Polymerase Chain Reaction (PCR-RFLP) technique, Mismatch Amplification Mutation Assay PCR (MAMA-PCR) technique, Matrix-Assisted Laser Desorption Ionization Time-of-Flight Mass Spectrometry (MassARRAY) technique, Phenol-Chloroform technique.

**Table 1:** Specific characteristics of the 13 included articles.

Reference	Country (Ethnicity)	Diagnostic criteria for depression	Severity of depression	Sample size				Gene	Genotype			CHD-D			CHD-nD			Depression			Healthy			Genotype method	HWE
				CHD-D	CHD-nD	Depression	Healthy																		
Nakatani et al., [31]	Japan (Asian)	NR	NR	861	942	NR	NR	5-HTTLPR	S/S	S/L	L/L	552	281	28	592	298	52	NR	NR	NR	NR	NR	NR	PCR	yes
Xia et al., 2006a [79]	China (Asian)	CCMD-III HAMD	depression	75	91	56	63	5-HTTLPR	S/S	S/L	L/L	38	26	11	27	36	28	28	21	7	18	25	20	PCR	yes
Xia et al., 2006b [85]	China (Asian)	CCMD-III	depression	70	70	NR	NR	5-HTTLPR	S/S	S/L	L/L	35	25	10	16	32	22	NR	NR	NR	NR	NR	NR	Phenol- Chloroform	Yes
Peng et al., 2017 (84)	China (Asian)	DSM-5 HAMD-17	depression	32	75	NR	NR	HSP70-rs2075799	G/G	G/A	A/A	18	12	2	48	26	1	NR	NR	NR	NR	NR	NR	PCR-RFLP	yes
Peng et al. 2018 (81)	China (Asian)	HAMD-17	depression	49	124	NR	NR	BDNFrs6265	G/G	G/A	A/A	3	30	16	37	57	30	NR	NR	NR	NR	NR	NR	PCR	yes
Wang 2020	China (Asian)	DSM-V PHQ-9	depression	123	147	NR	113	FKBP5-rs1360780	CC	CT	TT	60	56	7	74	65	8	NR	NR	NR	60	49	4	MassArray	Yes
								FKBP5-rs2817032	TT	TC	CC	63	52	8	79	56	12	NR	NR	NR	69	39	5		
								FKBP5-rs2817035	GG	GA	AA	52	65	6	67	75	5	NR	NR	NR	61	52	0		
								FKBP5-rs9296158	GG	GA	AA	53	56	14	59	65	23	NR	NR	NR	47	51	15		
								FKBP5-rs9470079	GG	GA	AA	71	44	8	75	58	14	NR	NR	NR	42	58	13		
								FKBP5-rs4713902	TT	TC	CC	62	50	11	83	59	5	NR	NR	NR	73	33	7		
								FKBP5-rs3800373	CC	CA	AA	35	82	6	37	100	10	NR	NR	NR	36	73	4		
Zhang et al., 2018 (87)	China (Asian)	NR	depression	412	453	NR	NR	microRNA146a-rs2910164	G/G	G/C	C/C	160	185	67	240	185	28	NR	NR	NR	NR	NR	NR	RT-qPCR	yes
Sara 2009	Italy (White)	BDI	MDD	29	70	NR	NR	BDNF-Val66Met	G/G	G/A	A/A	5	12	12	48	18	4	NR	NR	NR	NR	NR	NR	PCRRFLP	yes
Liu et al., 2014 (46)	China (Asian)	DSM-IVMD HAMD	depression	155	616	NR	NR	BDNF-rs16917204	G/G	G/C	C/C	48	69	38	158	330	128	NR	NR	NR	NR	NR	NR	MassARRAY platform	yes
								BDNF-rs6265	G/G	G/A	A/A	31	72	52	164	326	126	NR	NR	NR	NR	NR	NR		
								BDNF-rs7103873	G/G	G/C	C/C	99	47	9	358	222	36	NR	NR	NR	NR	NR	NR		
								BDNF-rs16917237	G/G	G/T	T/T	89	60	6	400	187	29	NR	NR	NR	NR	NR	NR		
								BDNF-rs56164415	C/C	C/T	T/T	131	24	0	528	88	0	NR	NR	NR	NR	NR	NR		
								BDNF-rs13306221	G/G	G/A	A/A	0	10	145	0	82	534	NR	NR	NR	NR	NR	NR		
								BDNF-rs2030323	G/G	G/T	T/T	43	82	30	182	286	148	NR	NR	NR	NR	NR	NR		
Han et al., 2020 (82)	China (Asian)	PHQ-9 DSM-IV	depression	49	165	NR	100	ACE2-rs2074192	C/C	C/T	T/T	21	15	13	59	52	54	NR	NR	NR	30	43	27	PCR	yes
								ACE2-rs714205	C/C	C/G	G/G	17	14	18	49	48	68	NR	NR	NR	27	41	32		
								ACE2-rs4646188	T/T	T/C	C/C	31	17	1	101	57	7	NR	NR	NR	66	29	5		
								ACE2-rs2285666	C/C	C/T	T/T	7	19	23	57	41	67	NR	NR	NR	32	44	24		
								MAS1-rs220721	C/C	C/T	T/T	3	12	34	23	38	104	NR	NR	NR	7	53	40		
								MAS1-rs220729	A/A	A/G	G/G	2	17	30	10	65	90	NR	NR	NR	3	40	57		
Golimbet et al., [83]	Moscow (White)	HAM-D-21 STAI	MDD	78	91	NR	121	IL-4–589	C/C	C/T	T/T	60	14	4	67	22	2	NR	NR	NR	72	41	8	PCR	yes
				78	78	91	NR	121	IL-6–174	G/G	G/C	C/C	29	43	6	31	46	14	NR	NR	NR	34	57			30
				78	78	91	NR	127	TNF-α–308	G/G	G/A	A/A	61	15	2	70	18	3	NR	NR	NR	96	29			2
				78	78	91	NR	127	CRP–717	A/A	A/G	G/G	49	27	2	56	28	7	NR	NR	NR	74	47			6
Han et al., 2019 (74)	China (Asian)	DSM-5 PHQ-9	depression	69	188	NR	107	SGK1-rs2758151	C/C	C/T	T/T	21	33	15	54	99	35	NR	NR	NR	30	54	23	PCRLDR	yes
								SGK1-rs1743963	A/A	A/G	G/G	10	24	35	31	97	60	NR	NR	NR	11	49	47		
								SGK1-rs9493857	A/A	A/G	G/G	3	18	48	12	67	109	NR	NR	NR	3	36	68		
								SGK1-rs1763509	G/G	A/G	A/A	2	19	48	21	70	97	NR	NR	NR	5	34	68		
								SGK1-rs9376026	C/C	C/T	T/T	48	20	1	131	51	6	NR	NR	NR	66	32	9		
								SGK1-rs9389154	G/G	A/G	A/A	10	38	21	43	87	58	NR	NR	NR	30	52	25		
Ma et al., 2011 (86)	China (Asian)	CCMD-III	depression	39	178	45	85	eNOS-G894T	G/G	G/T	T/T	31	8	0	120	57	1	36	9	0	67	18	0	PCR	yes

CHD-D, Coronary Heart Disease-Depression; CHD-nD, Coronary Heart Disease- non Depression.

Since only one article reported eNOS, HSP70, FKBP5, miR-146a, ACE2, MAS1, SGK1, IL-4–589, IL-6–174, TNF-α–308, and CRP–717 genes, a meta-analysis will not be conducted for these genes, we summarize the relationship of their allele frequencies in the patient and control groups in **Table 2**. Through analysis of the OR values of allele frequencies, an OR value greater than 1 indicates that the SNPs of that gene are risk factors for the development of coronary heart disease and depression, while an OR value less than 1 indicates that the SNPs of that gene are protective factors.

**Table 2:** The relationship between the allele frequencies of SNP reported in a single study and CHD-D.

Gene	Genotype	Sample (CHD-D/CHD-nD)	P-value	OR(95%CI)
HSP70	rs2075799G/A	32/75	0.30	1.45(0.72-2.92)
FKBP5	rs1360780C/T	123/147	0.82	1.05(0.72-1.52)
	rs2817032T/C	123/147	0.91	1.02(0.70-1.49)
	rs2817035G/A	123/147	0.55	1.12(0.77-1.62)
	rs9296158G/A	123/147	0.38	0.85(0.60-1.22)
	rs9470079G/A	123/147	0.21	0.78(0.53-1.15)
	rs4713902T/C	123/147	0.13	1.35(0.92-1.98)
	rs3800373C/A	123/147	0.54	0.90(0.63-1.27)
microRNA146a	rs2910164G/C	412/453	<0.01	1.74(1.42-2.14)
ACE2	rs2074192C/T	49/165	0.25	0.76(0.48-1.21)
	rs714205C/G	49/165	0.41	0.83(0.53-1.30)
	rs4646188T/C	49/165	0.65	0.88(0.50-1.54)
	rs2285666C/T	49/165	0.02	1.74(1.09-2.80)
MAS1	rs220721C/T	49/165	0.15	1.52(0.86-2.68)
	rs220729A/G	49/165	0.38	1.27(0.74-2.19)
IL-4	589C/T	78/91	0.96	0.99(0.53-1.82)
IL-6	174G/C	78/91	0.31	0.79(0.51-1.24)
TNF-α	308G/A	78/91	0.78	0.91(0.48-1.74)
CRP	717A/G	78/91	0.48	0.83(0.49-1.39)
SGK1	rs2758151C/T	69/188	0.89	1.03(0.70-1.52)
	rs1743963A/G	69/188	0.03	1.57(1.04-2.36)
	rs9493857A/G	69/188	0.10	1.52(0.92-2.50)
	rs1763509G/A	69/188	<0.01	2.12(1.29-3.49)
	rs9376026C/T	69/188	0.83	0.94(0.55-1.60)
	rs9389154G/A	69/188	0.42	1.18(0.79-1.74)
eNOS	G894TG/T	39/178	0.17	0.58(0.26-1.26)

CHD-D, Coronary Heart Disease-Depression; CHD-nD, Coronary Heart Disease- non Depression.

For the 5-HTTLPR genotype and the BDNF genotype at rs6265 locus, we will use Review Manager 5.4 software to calculate the effect sizes. We will analyze the 5-HTTLPR gene using different genetic models, including the allele model (S vs L), the dominant model (LS+SS vs LL), the recessive model (SS vs LL+LS), the co-dominant model (LS vs LL; SS vs LL), and the homozygous model (SS vs LL). For the BDNF rs6265 gene, we will analyze it using different genetic models, including the allele model (A vs G), the dominant model (GA+AA vs GG), the recessive model (AA vs GG+GA), the co-dominant model (GA vs GG; AA vs GG), and the homozygous model (AA vs GG) refer to **Supplementary Table 1** for specific study characteristics. We conducted subgroup analysis based on racial factors for the genes 5-HTTLPR and BDNF rs6265 to explore whether race influences the final effect size. We performed a sensitivity analysis using Stata 16.0 software to assess the stability of the results for each genetic model.

### 3.2 Meta-analysis results

#### 3.2.1 5-HTTLPR polymorphism

**Figure 2:**
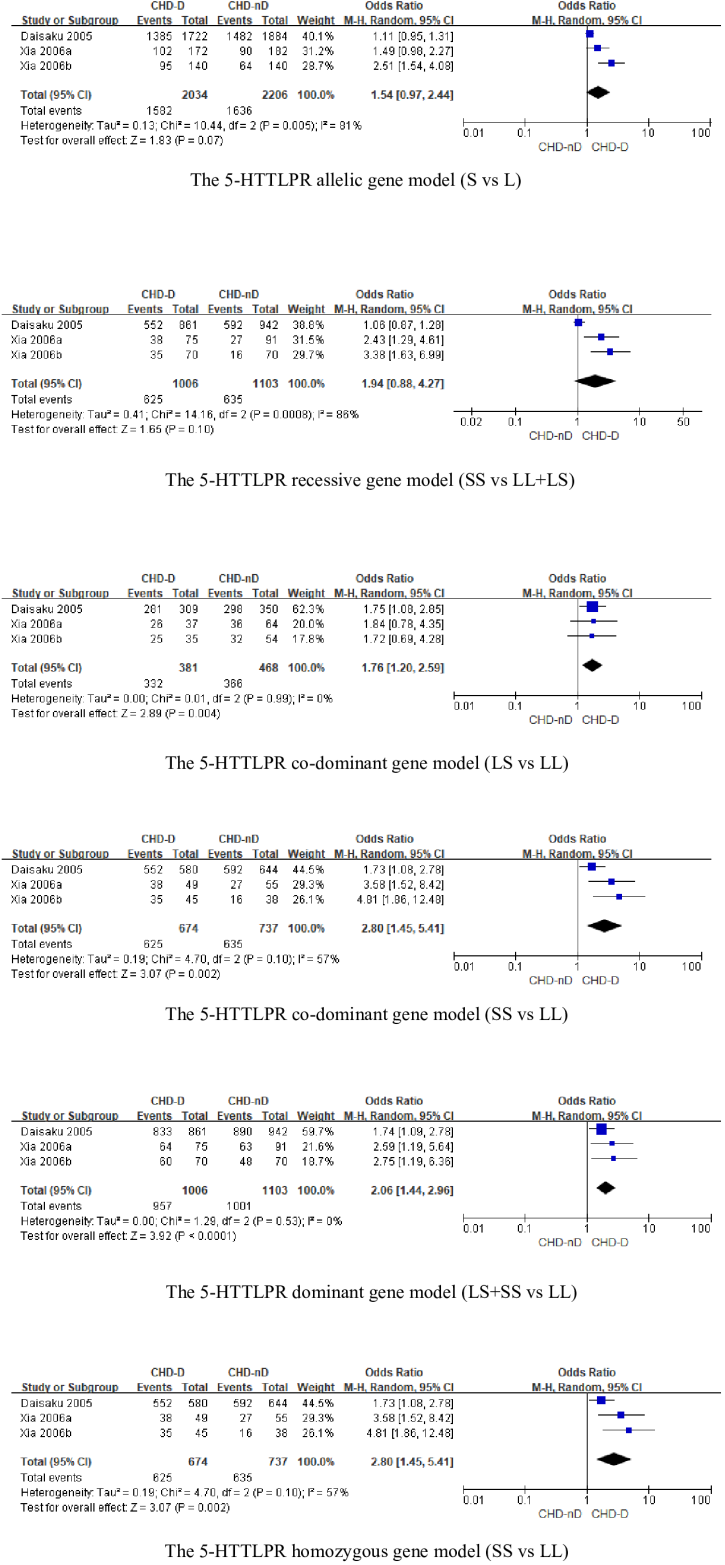
The 5-HTTLPR genotype.

The 5-HTTLPR genotype was included in a total of 3 articles, involving 1006 patients with CHD-D and 1103 patients with CHD-nD, see **Figure 2**. The results showed that the allelic and recessive models of the 5-HTTLPR gene were not statistically significant. However, the co-dominant model (LS vs LL: OR 1.76, 95%CI 1.20 to 2.59; SS VS LL: OR 2.80, 95%CI 1.45 to 5.41), dominant model (LS+SS VS LL: OR 2.06, 95%CI 1.44 to 2.96), and homozygous model (SS vs LL: OR 2.80 95%CI 1.45 to 5.41) were statistically significant, indicates that the SNPs of the 5-HTTLPR gene are risk factors for the development of coronary heart disease and depression.

#### 3.2.2 BDNF rs6265 polymorphism

**Figure 3:**
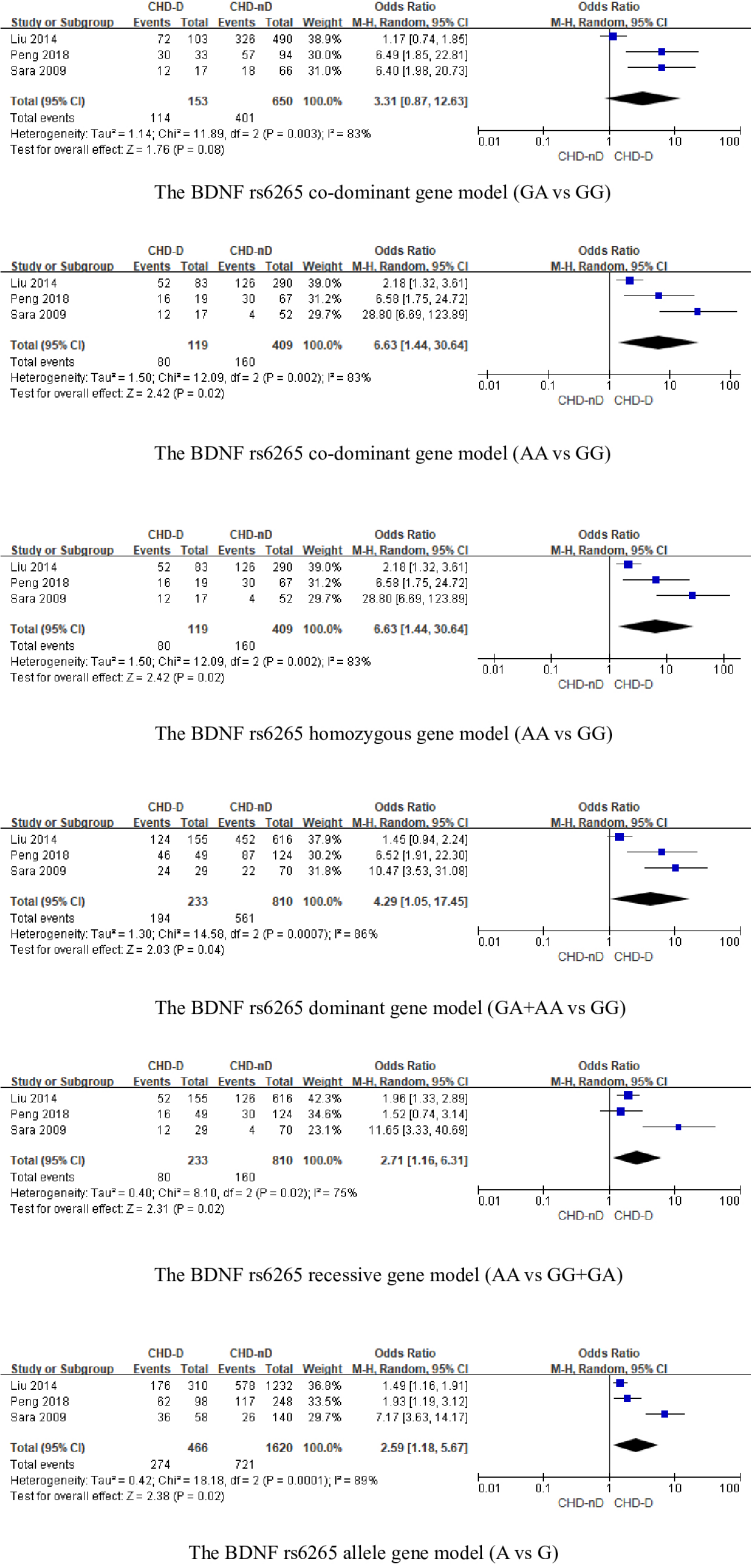
The BDNFrs6265 genotype.

The BDNFrs6265 gene was included in a total of 3 articles, involving 233 patients with CHD-D and 810 patients with CHD-nD, see **Figure 3**. The results showed that the co-dominant gene model (GA vs GG) of the BDNF rs6265 gene was not statistically significant. However, the co-dominant gene model (AA vs GG: OR 6.63, 95% CI 1.44 to 30.64) had statistical significance. Furthermore, the homozygous gene model (AA vs GG: OR 6.63, 95% CI 1.44 to 30.64), the dominant gene model (GA+AA vs GG: OR 4.29, 95% CI 1.05 to 17.45), the recessive gene model (AA vs GG+GA: OR 2.71, 95% CI 1.16 to 6.31), and the allele model (A vs G: OR 2.59, 95% CI 1.18 to 5.67) all showed statistical significance, indicates that the SNPs of the BDNFrs6265 gene are risk factors for the development of coronary heart disease and depression.

#### 3.2.3 The allele frequency of the 11 SNPs

In this study, the relationship between the allele frequencies of the 11 SNPs (eNOS, HSP70, FKBP5, miR-146a, ACE2, MAS1, SGK1, IL-4–589, IL-6–174, TNF-α–308, CRP–717) which have only been reported in a single study, the allele frequency data for these SNPs were compiled and organized in **Table 2**. The results indicate that there is a statistically significant difference in the allele frequencies of rs2910164 genotype in the microRNA146a gene, rs2285666 genotype in the ACE2, as well as rs1743963 and rs1763509 genotypes in the SGK1 gene between the patient group (CHD-D) and the control group (CHD-nD). However, there is no statistically significant difference in the allele frequencies of the remaining genes. This indicates that the SNPs of rs2910164 in the microRNA146a gene, the SNPs of rs2285666 in the ACE2 gene, the SNPs of rs1743963 and rs1763509 in the SGK1 gene are risk factors for the development of coronary heart disease and depression.

### 3.3 Subgroup analysis

**Figure 4:**
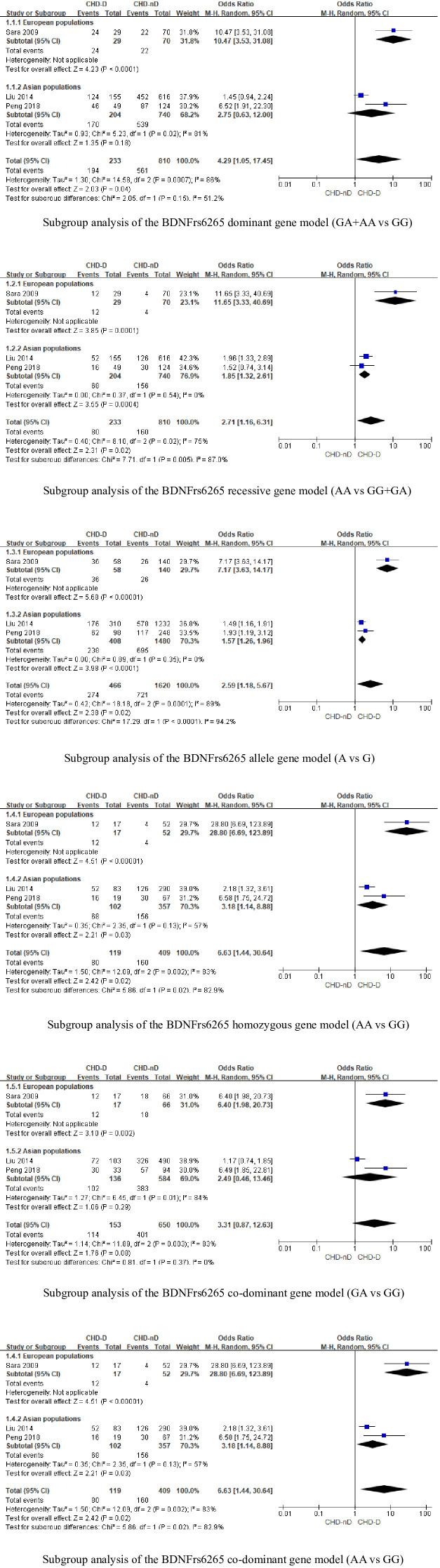
Subgroup analysis of BDNFrs6265 gene.

We conducted an ethnicity-based subgroup analysis of studies involving the 5-HTTLPR and BDNFrs6265 genes. However, since the three literature articles involving the 5-HTTLPR gene were restricted to Asian populations, we will not proceed with subgroup analysis for this gene. On the other hand, we performed a subgroup analysis of three literature articles involving the BDNFrs6265 gene, see **Figure 4**. The results showed that in the dominant model (GA+AA vs GG: OR 10.47, 95%CI 3.53 to 31.08) and the co-dominant model (GA vs GG: OR 6.40, 95%CI 1.98 to 20.73), European populations are more susceptible to an increased risk of comorbid coronary heart disease and depression compared to Asian populations. This difference is statistically significant.

### 3.4 Sensitivity analysis and publication bias

**Figure 5:**
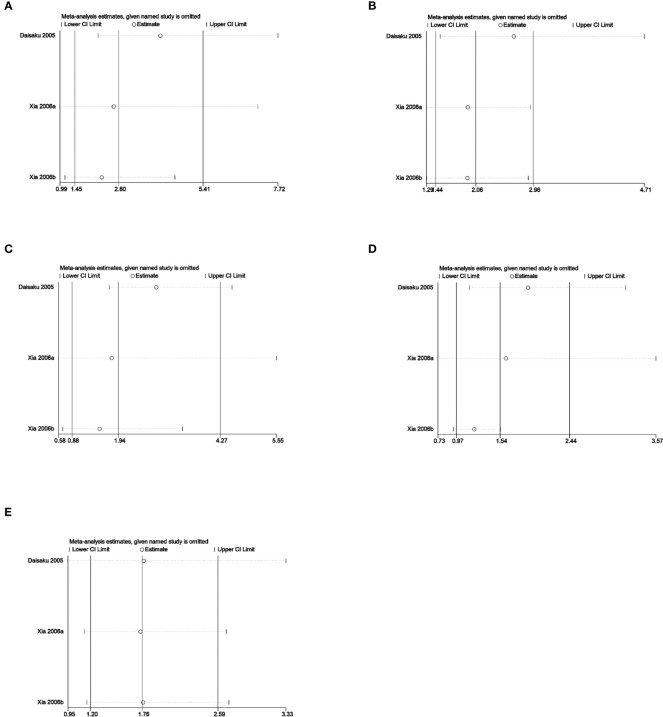
**(A)** Sensitivity analysis of the 5-HTTLPR homozygous model (SS vs LL); **(B)** Sensitivity analysis of the 5-HTTLPR dominant model (LS+SS vs LL); **(C)** Sensitivity analysis of the 5-HTTLPR recessive model (SS vs LL+LS); **(D)** Sensitivity analysis of the 5-HTTLPR allele model (S vs L); **(E)** Sensitivity analysis of the 5-HTTLPR co-dominant model (LS vs LL).

**Figure 6:**
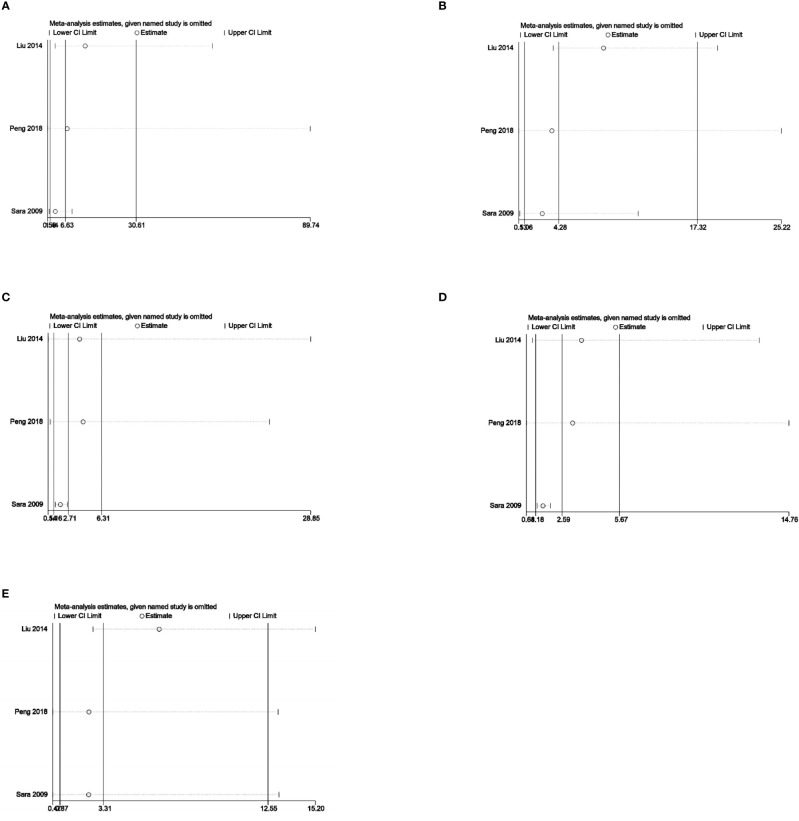
**(A)** Sensitivity analysis of the BDNFrs6265 homozygous model (AA vs GG); **(B)** Sensitivity analysis of the BDNFrs6265 dominant model (GA+AA vs GG); **(C)** Sensitivity analysis of the BDNFrs6265 recessive model (AA vs GG+GA); **(D)** Sensitivity analysis of the BDNFrs6265 allele model (A vs G); **(E)** Sensitivity analysis of the BDNFrs6265 co-dominant model (GA vs GG).

Sensitivity analysis was used to analyze whether the ratio-ratio (OR) value of each genotype had a significant effect on the combined OR value to explore the stability of the results. The results showed that even after sequentially excluding individual data, the allelic model, dominant model, recessive model, homozygous model, and co-dominant model of both the 5-HTTLPR and BDNF rs6265 genes yielded similar results to the combined OR value. The indicates that the results of this meta-analysis are stable, see **Figures 5**, **6**. Due to the limited number of studies on the 5-HTTLPR and BDNF genes, it is not possible to determine the symmetry of the funnel plot using Begg’s funnel plot and Egger’s test. Therefore, we will not assess publication bias.

## 4 Discussion

This article is the first meta-analysis of all currently known candidate genes for depression in combination with CHD. Many clinical studies have shown that genetic information can help predict the development of diseases, select the most effective therapeutic interventions, and reduce complications (96). Exploring the comorbid genes of CHD and depression can help clinicians choose the best treatment drugs and other therapies for patients, reduce the economic burden and time cost of patients, and avoid medical waste at the same time. This research also inspires the development of new drugs. If the data is of poor quality, partial results may be obtained, and future research suggestions will be provided.

This study investigated the potential relationship between 13 SNPs and the comorbidity of coronary heart disease and depression. The results of the study showed that SNPs of eNOS, HSP70, FKBP5, miR-146a, ACE2, MAS1, SGK1, IL-4–589, IL-6–174, TNF-α–308, CRP–717, 5-HTTLPR, and BDNF genes are associated with the comorbidity of coronary heart disease and depression. Among them, only one study reported the association of SNPs of eNOS, HSP70, FKBP5, miR-146a, ACE2, MAS1, SGK1, IL-4–589, IL-6–174, TNF-α–308, and CRP–717 genes, so meta-analysis could not be performed. The results showed that the allele frequencies of rs2910164 in the microRNA146a gene, rs2285666 in the ACE2 and rs1743963 and rs1763509 in the SGK1 gene were statistically different between the Case and control group. In contrast, the allele frequencies of the other genes showed no statistical difference between the groups. The indicates that the SNPs of rs2910164 in the microRNA146a gene, rs2285666 in the ACE2 and rs1743963 and rs1763509 in the SGK1 gene are risk factors for the development of coronary heart disease and depression. A total of three studies were included in the 5-HTTLPR gene, with 1006 cases in the CHD-D group and 1103 cases in the CHD-nD group. A total of three studies were included in the BDNF gene, with 233 cases in the CHD-D group and 810 cases in the CHD-nD group. Meta-analysis showed that there were statistically significant differences in the co-dominant model (OR 2. 80), dominant model (OR 2. 06), and homozygous model (OR 2. 80) of the 5-HTTLPR gene between the CHD-D group and CHD-nD group, indicating that the SNPs of the 5-HTTLPR gene are associated with the risk of developing coronary heart disease and depression. There were also statistically significant differences in the co-dominant model (AA vs GG: OR 6. 63), homozygous model (OR 6. 63), dominant model (OR 4. 29), recessive model (OR 2. 71), and allele model (OR 2. 59) of the BDNF gene between the CHD-D group and CHD-nD group, indicating that the SNPs of the BDNF gene are associated with the risk of developing coronary heart disease and depression.

In addition, ethnicity-based subgroup analyses of the 5-HTTLPR and BDNF genes were performed. The results showed that the dominant model (GA+AA vs GG: OR 10. 47) and co-dominant model (GA vs GG: OR 6. 40) of the BDNF gene were more likely to increase the risk of developing coronary heart disease and depression in the European population compared to the Asian population. The studies that included the 5-HTTLPR gene were conducted in Asian populations, so subgroup analyses were not feasible. Sensitivity analysis of the 5-HTTLPR and BDNF genes in the included studies showed stable results. Since the number of studies reporting SNPs of the 5-HTTLPR and BDNF genes was less than 10, Begg’s funnel plot and Egger’s test were not conducted to assess publication bias.

This meta-analysis still has limitations. We comprehensively reviewed the relevant studies on the impact of SNPs on comorbid depression in coronary heart disease, but the number of studies is still limited. There were only three studies of the 5-HTTLPR gene and three studies of the BDNF gene. Moreover, the studies on the 5-HTTLPR gene are concentrated in the years 2005 and 2006, which may indicate potential publication bias.

## 5 Conclusion

In summary, this study demonstrates that SNPs of the microRNA146a gene at rs2910164, ACE2 gene at rs2285666 and the SGK1 gene at rs1743963 and rs1763509, and the SNPs at the 5-HTTLPR and BDNF gene loci are associated with the onset of comorbid depression in coronary heart disease. Subgroup analysis of the three included studies on the BDNF gene at rs6265 revealed that the SNPs at this locus in the European population are more likely to increase the risk of comorbid depression in coronary heart disease (dominant model GA+AA vs GG: OR 10. 47, 95%CI 3. 53 to 31. 08; co-dominant model GA vs GG: OR 6. 40, 95%CI 1. 98 to 20. 73). Sensitivity analysis of the studies reporting on the 5-HTTLPR and BDNF gene at rs6265 showed stable results. However, due to the limited number of studies included for the 5-HTTLPR and BDNF rs6265 gene loci, potential publication bias may exist. We suggest that future studies should focus on examining the effects of SNPS on combined depression in coronary artery disease, especially targeting the 5-HTTLPR and BDNF genes of rs6265.

## Data availability statement

The original contributions presented in the study are included in the article/**Supplementary Material**. Further inquiries can be directed to the corresponding authors.

## Author contributions

JZ: Writing – original draft. LG: Writing – review & editing. DK: Writing – original draft, Writing – review & editing. GY: Writing – original draft.

## Glossary

**Table:**

CHD	Coronary heart disease
HPA	hypothalamus-pituitary-adrenal
5-HTT	5-hydroxytryptamine transporter
5-HT	serotonergic
5-HTTLPR	5-HT transporter linked promoter region
5-HT2AR	Serotonin 2A receptor
5-HT2CR	serotonin 2C receptor
GPCR	G-protein-coupled receptor
MDD	Major depressive disorder
APLN	Apelin
BDNF	Brain-derived neurotrophic factor
ECs	endothelial cells
ApoE	Apolipoprotein E
FKBP5	FK-506 binding protein 51
hsp90	heat shock protein 90
GR	glucocorticoid receptor
NR3C1	Nuclear receptor subfamily 3 group C member1
SGK1	Serum/glucocorticoid-regulated kinase 1
IGF1	insulin-like growth factor 1
PAI-1	Plasminogen activator inhibitor-1
tPA	tissue plasminogen activator
uPA	urokinase plasminogen activators
SERPINE1	plasminogen activator inhibitor-1 gene
PRISMA-P	Preferred Reporting Items for Systematic Review and Meta-Analysis Protocols
PROSPERO	published on the International Prospective Register of Systematic Reviews
CENTRAL	Cochrane Central Register of Controlled Trials
CNKI	the China National Knowledge Infrastructure
CQVIP	Chongqing VIP
SinoMed	China Biomedical Literature Service System
ChiCTR	the Chinese Clinical Trial Registry
HuGENavigator	Human Genomic Epidemiology Navigator
MeSH	medical subject headings
SNPs	single nucleotide polymorphisms
CAD	coronary artery disease
CVD	cardio vascular disease
ACS	acute coronary syndrome
MI	myocardial infarction
HWE	Hardy-Weinberg Equilibrium
NOS	the Newcastle-Ottawa Scalel
