# Supplementary material for: The effect of single nucleotide polymorphisms on depression in combination with coronary diseases: a systematic review and meta-analysis
Source: Front Endocrinol (Lausanne). 2024 Apr 30;15:1369676. doi: 10.3389/fendo.2024.1369676 (PMC11091366; doi:10.3389/fendo.2024.1369676)
Supplement: Supplementary file 1 [file Table_1.docx]

Supplementary Table 1. Genetic characterization of the 5-HTTLPR and BDNF genes included in the literature

| Author | Country | Diagnostic criteria for depression | Sample size | | | | | Gene | CHD-D | | | | | CHD-nD | | | | | Depression | | | | | Healthy | | | | |
| --- | --- | --- | --- | --- | --- | --- | --- | --- | --- | --- | --- | --- | --- | --- | --- | --- | --- | --- | --- | --- | --- | --- | --- | --- | --- | --- | --- | --- |
|  |  |  | CHD-D | CHD-nD | Depression | | Healthy |  | S/S | S/L | L/L | S | L | S/S | S/L | L/L | S | L | S/S | S/L | L/L | S | L | S/S | S/L | L/L | S | L |
| Xia et al. | China | CCMD-Ⅲ  HAMD | 75 | 91 | 56 | 63 | | 5-HTTLPR | 38 | 26 | 11 | 102 | 70 | 27 | 36 | 28 | 90 | 92 | 28 | 21 | 7 | 77 | 35 | 18 | 25 | 20 | 61 | 65 |
| Xia et al. | China | CCMD-Ⅲ | 70 | 70 | NR | NR | | 5-HTTLPR | 35 | 25 | 10 | 95 | 45 | 16 | 32 | 22 | 64 | 76 | NR | NR | NR | NR | NR | NR | NR | NR | NR | NR |
| Daisaku et al. | Japan | NR | 861 | 942 | NR | NR | | 5-HTTLPR | 552 | 281 | 28 | 1385 | 337 | 592 | 298 | 52 | 1482 | 402 | NR | NR | NR | NR | NR | NR | NR | NR | NR | NR |

| Author | Country | Diagnostic criteria for depression | Sample size | | | | | Gene | CHD-D | | | | | CHD-nD | | | | | Depression | | | | | Healthy | | | | |
| --- | --- | --- | --- | --- | --- | --- | --- | --- | --- | --- | --- | --- | --- | --- | --- | --- | --- | --- | --- | --- | --- | --- | --- | --- | --- | --- | --- | --- |
|  |  |  | CHD-D | CHD-nD | Depression | | Healthy |  | G/G | G/A | A/A | G | A | G/G | G/A | A/A | G | A | G/G | G/A | A/A | G | A | G/G | G/A | A/A | G | A |
| Sara et al. | Italy | BDI | 29 | 70 | NR | NR | | BDNF Val66Met | 5 | 12 | 12 | 22 | 36 | 48 | 18 | 4 | 114 | 26 | NR | NR | NR | NR | NR | NR | NR | NR | NR | NR |
| Peng et al. | China | HAMD-17 | 49 | 124 | NR | NR | | BDNF  rs6265 | 3 | 30 | 16 | 36 | 62 | 37 | 57 | 30 | 131 | 117 | NR | NR | NR | NR | NR | NR | NR | NR | NR | NR |
| Liu et al. | China | DSM-IVMD  HAMD | 155 | 616 | NR | NR | | BDNF rs16917204 | GG | GC | CC | G | C | GG | GC | CC | G | C | NR | NR | NR | NR | NR | NR | NR | NR | NR | NR |
|  |  |  |  |  |  |  |  | BDNF  rs6265 | 31 | 72 | 52 | 134 | 176 | 164 | 326 | 126 | 654 | 578 | NR | NR | NR | NR | NR | NR | NR | NR | NR | NR |
|  |  |  |  |  |  |  |  | BDNF  rs7103873 | GG | GC | GG | G | C | GG | GC | CC | G | C | NR | NR | NR | NR | NR | NR | NR | NR | NR | NR |
|  |  |  |  |  |  |  |  | BDNF  rs16917237 | GG | GT | TT | G | T | GG | GT | TT | G | T | NR | NR | NR | NR | NR | NR | NR | NR | NR | NR |
|  |  |  |  |  |  |  |  | BDNF  rs56164415 | CC | CT | TT | C | T | CC | CT | TT | C | T | NR | NR | NR | NR | NR | NR | NR | NR | NR | NR |
|  |  |  |  |  |  |  |  | BDNF  rs13306221 | 0 | 10 | 145 | 10 | 300 | 0 | 82 | 534 | 82 | 1150 | NR | NR | NR | NR | NR | NR | NR | NR | NR | NR |
|  |  |  |  |  |  |  |  | BDNF  rs2030323 | GG | GT | TT | G | T | GG | GT | TT | G | T | NR | NR | NR | NR | NR | NR | NR | NR | NR | NR |

CHD-D Coronary Heart Disease-Depression；CHD-nD Coronary Heart Disease- non Depression
